# Receipt of Posthospitalization Care Training Among Medicare Beneficiaries’ Family Caregivers

**DOI:** 10.1001/jamanetworkopen.2021.1806

**Published:** 2021-03-16

**Authors:** Julia G. Burgdorf, Chanee D. Fabius, Catherine Riffin, Jennifer L. Wolff

**Affiliations:** 1 Department of Health Policy & Management, Johns Hopkins Bloomberg School of Public Health, Baltimore, Maryland; 2 Department of Internal Medicine, Weill Cornell Medicine, New York, New York

## Abstract

**Importance:**

Medicare conditions of participation require hospitals to provide training to family and unpaid caregivers when their support is necessary to enact the postdischarge care plan. However, caregivers often report feeling unprepared for this role.

**Objective:**

To describe the characteristics of caregivers who assist with posthospitalization care transitions and assess the prevalence of and factors associated with receipt of adequate transitional care training.

**Design, Setting, and Participants:**

This cross-sectional study analyzed data from the 2017 National Health and Aging Trends Study and its linked National Study of Caregiving, surveys of Medicare beneficiaries and their family and unpaid caregivers. The present study included family caregivers for community-living Medicare beneficiaries 65 years or older with disabilities. Data analysis was performed from June to September 2020.

**Main Outcomes and Measures:**

Characteristics of family caregivers by whether they assisted during a posthospitalization care transition in the year preceding the survey interview. Unweighted frequencies and weighted percentages, as well as the results of weighted Pearson and Wald tests for differences between groups, are reported. Receipt of the training needed to manage the older adult’s posthospitalization care transition (hereafter referred to as adequate transitional care training) as a function of individual caregiver characteristics was modeled using multivariable, weighted logistic regression.

**Results:**

Of 1905 family caregivers, 618 (58.9%) were 60 years or older, 1288 (63.8%) were female, and 796 (41.7%) assisted with a posthospitalization care transition. Those who assisted with a posthospitalization care transition were more likely to report experiencing financial (154 [18.3%] vs 123 [10.1%]; *P* < .001), emotional (344 [41.3%] vs 342 [31.1%]; *P* < .001), and physical (200 [22.2%] vs 170 [14.6%]; *P* = .001) difficulty associated with caregiving. Among caregivers who assisted during a posthospitalization care transition, 490 (59.1%) reported receiving adequate transitional care training. Caregivers were less likely to report receiving adequate training if they assisted an older adult who was female (316 [62.3%] vs 227 [73.2%]; *P* = .02), Black (163 [14.0%] vs 121 [19.8%]; *P* = .02), or enrolled in Medicaid (127 [21.2%] vs 90 [31.9%]; *P* = .01). After adjusting for older adult characteristics, caregivers were half as likely to report receiving adequate training if they were Black (adjusted odds ratio [aOR], 0.52; 95% CI, 0.31-0.89) or experienced financial difficulty (aOR, 0.50; 95% CI, 0.31-0.81). Caregivers were more than twice as likely to report receiving adequate training if they were female (aOR, 2.44; 95% CI, 1.65-3.61) or spoke with the older adult’s clinician about his or her care in the past year sometimes or often vs never (aOR, 1.93; 95% CI, 1.19-3.12).

**Conclusions and Relevance:**

In this cross-sectional study, caregivers were less likely to receive adequate transitional care training if they were Black; experienced financial difficulty; or cared for a Black, female, or Medicaid-enrolled older adult. These findings suggest that changes to the discharge process, such as using standardized caregiver assessments, may be necessary to ensure equitable support of family caregivers.

## Key Points

**Question:**

Does receipt of adequate training related to managing older adults’ posthospitalization care vary by family caregiver and older adult characteristics?

**Findings:**

In a cross-sectional study of 1905 caregivers assisting an older adult after hospital discharge, 59.1% reported receiving the training that they needed. Caregivers were less likely to receive the training that they needed if they were Black; experienced financial difficulty; or cared for a Black, female, or Medicaid-enrolled older adult.

**Meaning:**

The findings suggest concerning disparities in caregivers’ receipt of needed training when managing posthospitalization care transitions for older adults.

## Teaser

This cross-sectional study assesses the prevalence and factors associated with receipt of adequate transitional care training among caregivers of older adults undergoing posthospitalization care transition.

## Introduction

Care transitions refer to the movement of patients between care settings in accordance with changing health status and needs.^1^ Care transitions are associated with myriad challenges for clinicians and patients. Older adults in particular are at increased risk for medical errors, communication gaps, and unplanned readmission during posthospitalization care transitions.^2,3,4^ The Centers for Medicare & Medicaid Services has implemented new policies and payment models aimed at improving posthospitalization care transition quality, including the Hospital Readmissions Reduction Program, Bundled Payments for Care Improvement, and transitional care management billing codes.^5,6,7^

Family caregivers are often involved in supporting older adults throughout posthospitalization care.^8,9,10^ Family caregivers’ actions, including assisting with medical and nursing tasks,^11^ are associated with health care utilization and outcomes,^12,13,14,15,16,17,18,19^ and effective clinician-caregiver communication is associated with reduced patient postdischarge resource use and decreased risk of unplanned readmission.^8,20,21,22^ The Joint Commission has identified patient and family caregiver engagement as 1 of 7 foundations for safe and effective care transitions.^23^

Emerging reports in the literature suggest that training of family caregivers is associated with reduced caregiver burden and improved care quality and patient outcomes.^8,9,24,25^ Caregivers report needing additional instruction related to medical and nursing tasks.^26^ However, fewer than 1 in 10 caregivers report receiving role-related training,^27^ and caregivers commonly report learning how to manage medical and nursing tasks on their own without guidance or instruction from formal health care professionals.^26^ Training may be especially important during posthospitalization care transitions. Patients and family caregivers consistently report feeling unprepared for hospital discharge,^28,29,30^ and caregivers often assume greater responsibility for medical and nursing tasks while they are providing posthospitalization care.^31^

Interest in expanding health care professional–led support of family caregivers during care transitions has motivated the development and dissemination of transitional care models that prioritize caregiver education in the US.^32,33^ In addition, 40 states have passed the Caregiver Advise, Record, and Enable (CARE) Act to increase hospital support and education of caregivers during the hospital discharge process.^34^ To our knowledge, no previous literature presents nationally representative information regarding the association between caregiver characteristics and receipt of training related to assisting with a posthospitalization care transition. Such information could prove to be useful in monitoring efforts to improve access to training related to management of posthospitalization care.

We used linked, nationally representative surveys of older adults and their family caregivers to describe the characteristics of caregivers who assisted older adult patients during a posthospitalization care transition, the proportion of caregivers who reported receiving the training that they needed to manage this care (hereafter referred to as adequate transitional care training), and factors associated with receipt of this training. Findings are relevant to ongoing efforts by researchers, clinicians, and policy makers to improve postdischarge outcomes for older adults and to expand hospital-based support of family caregivers.

## Methods

### Sample

This cross-sectional study used data from the 2017 National Health and Aging Trends Study (NHATS) and National Study of Caregiving (NSOC), 2 linked national surveys that collect comprehensive information from both older adults and their family caregivers. The NHATS is a nationally representative survey of Medicare beneficiaries 65 years or older in which in-person interviews are used to collect measures of health status, function, and reliance on family caregiving. The NSOC is a companion telephone survey of up to 5 family and unpaid caregivers identified by NHATS participants. The NSOC was first fielded in 2011, with additional waves in 2015 and 2017. This study was deemed not human subjects research by the Johns Hopkins Bloomberg School of Public Health institutional review board because all data are deidentified and publicly available. Informed consent was obtained from survey participants during data collection for the NHATS and NSOC; these data-gathering activities were wholly separate from analyses for this study. The study followed the Strengthening the Reporting of Observational Studies in Epidemiology (STROBE) reporting guideline.

To be eligible for the NSOC, a caregiver must have assisted a community-living older adult with mobility, personal care, or household activities for health and function reasons, or assisted an older adult who lived in a residential care facility. This study focused on caregivers of community-living older adults given that supports available in residential care facilities could substitute for or otherwise affect the need for family caregiving after hospital discharge. Of the 2652 caregivers who completed the 2017 NSOC, the analytic sample for this study included 1905 caregivers who were assisting a community-living older adult at the time of survey.

### Measures

Our measure of whether caregivers assisted during a posthospitalization care transition was based on caregiver responses to the question “In the last year, did you help [NHATS participant] after an overnight stay at a hospital?” Among those assisting during posthospitalization transitions, a binary indicator of receiving needed transitional care training was constructed. Caregivers were defined as having reported receiving adequate transitional care training if they responded affirmatively to the question “Did medical providers at the hospital give you the training you needed to manage [NHATS participant’s] post-hospital care?”

Other caregiver characteristics included sociodemographic data (sex, race/ethnicity, and educational attainment); experiences of financial, emotional, and physical difficulty owing to caregiving; caregiving circumstances (being a sole caregiver, receiving payment for providing care, caregiving for ≥5 years, mean number of hours of care provided per month, and types of assistance provided); and interactions with the health care system (frequency of discussing older adult’s care with a clinician in the past year). Race/ethnicity was measured using caregiver self-reports of primary race/ethnicity. Fewer than 10% of respondents identified as Hispanic or another race/ethnicity. Therefore, we created a variable reflecting whether an individual identified as non-Hispanic White, non-Hispanic Black, or other race/ethnicity. Caregiving-related difficulty refers to an affirmative response to the question “Is helping [NHATS participant] [financially/emotionally/physically] difficult for you?” Types of assistance included mobility (ambulation within the home, transferring, and leaving the home), personal care (eating, bathing, toileting, and dressing), health care (managing medications, performing medical tasks, administering shots, and providing wound care), and health care system navigation (making health care appointments and coordinating care).

We measured older adults’ sociodemographic characteristics, including age, sex, race/ethnicity, and Medicaid enrollment, and characteristics associated with the need for caregiver assistance and/or caregiving experiences, including self-reported health status, dementia, and postdischarge destination.^9,35^ Race/ethnicity was measured using older adult self-reports of primary race/ethnicity. Because our sample was limited to Medicare beneficiaries 65 years or older, those who were enrolled in Medicaid represented the dual-eligible population. Dementia refers to a composite measure from self-reported physician diagnosis of Alzheimer disease or dementia, proxy respondent responses to a dementia screening tool, and older adult performance on cognitive tests in the NHATS, as described previously by Kasper et al.^36^ All older adult characteristics were drawn from NHATS except for postdischarge destination. Postdischarge destination was drawn from NSOC and refers to whether the older adult was transitioning to an institution or to home (with or without home health care) after hospital discharge.

### Statistical Analysis

Data analysis was performed form June to September 2020. First, we described the characteristics of family caregivers by whether they assisted during a posthospitalization care transition in the year preceding the survey interview. We present unweighted frequencies and weighted percentages as well as the results of weighted Pearson and Wald tests for differences between groups. For all analyses, statistical significance was determined by α < .05 in 2-sided hypothesis tests.

Next, we limited our analyses to caregivers who assisted with posthospitalization transitions and comparatively examined characteristics of caregivers (and their care recipients) by whether the caregiver received adequate transitional care training. We tested for differences between groups using weighted Pearson and Wald tests. We then modeled receipt of adequate transitional care training as a function of individual caregiver characteristics using multivariable, weighted logistic regression. We adjusted for older adult age, sex, self-reported health status, dementia, and postdischarge destination. We excluded older adult race and Medicaid enrollment from these models because these variables were collinear with 2 caregiver characteristics of interest (race/ethnicity and financial difficulty, respectively). All analyses used survey weights and design variables to account for complex, nonprobability sampling and to produce nationally representative estimates. Analyses were conducted using Stata, version 14 (StataCorp LLC).

## Results

Of 1905 (weighted n = 17 268 357) family caregivers, 618 (58.9%) were 60 years or older and 1288 (63.8%) were female. A total of 796 caregivers (41.7%; 95% CI, 38.4%-45.1%) helped an older adult after an overnight hospital stay within the previous 12 months. Compared with those who did not assist an individual with a posthospitalization care transition, those who assisted were more likely to be Black (280 [17.5%] vs 308 [12.3%]; *P* = .01) and to experience financial (154 [18.3%] vs 123 [10.1%]; *P* < .001), emotional (344 [41.3%] vs 342 [31.1%]; *P* < .001), and physical (200 [22.2%] vs 170 [14.6%]; *P* = .001) difficulty associated with caregiving (Table 1). Compared with caregivers who did not assist with a posthospitalization care transition, those who did provided more hours of care per month (mean [SE], 82.8 [6.3] hours vs 55.8 [3.5] hours; *P* < .001) and were more likely to assist with mobility (676 [85.8%] vs 794 [67.4%]; *P* < .001), personal care (570 [69.7%] vs 577 [44.9%]; *P* < .001), health care tasks (562 [67.1%] vs 602 [48.2%]; *P* < .001), and health care system navigation (539 [63.3%] vs 519 [37.5%]; *P* < .001).

**Table 1.** Characteristics of Family Caregivers Who Did vs Did Not Assist With a Posthospitalization Care Transition in the Past Year and Characteristics of Their Care Recipients^a^

Characteristic	Individuals, No. (%)^b^		*P* value^c^
	Assisted during transition (n = 796)	Did not assist during transition (n = 1109)	
**Caregiver characteristics**			
Female	555 (64.1)	733 (63.6)	.87
Race/ethnicity			
White	417 (65.4)	669 (73.3)	.01
Black	280 (17.5)	308 (12.3)	
Other	99 (17.1)	132 (14.4)	
Educational attainment			
High school or less	307 (40.7)	405 (36.1)	.20
Some college	459 (59.3)	677 (63.9)	
Experiences of burden			
Financial difficulty	154 (18.3)	123 (10.1)	<.001
Emotional difficulty	344 (41.3)	342 (31.1)	<.001
Physical difficulty	200 (22.2)	170 (14.6)	.001
**Caregiving circumstances**			
Sole caregiver	196 (27.2)	330 (34.2)	.05
Relationship to older adult			
Spouse	185 (25.7)	242 (22.2)	.006
Child	440 (50.8)	571 (45.4)	
Other	171 (23.5)	296 (32.4)	
Paid for caregiving	172 (19.5)	165 (14.0)	.01
Caregiver for ≥5 y	241 (52.7)	315 (52.5)	.96
Caregiving h/mo, mean (SE)	82.8 (6.3)	55.8 (3.5)	<.001
Type of assistance			
Mobility	676 (85.8)	794 (67.4)	<.001
Personal care	570 (69.7)	577 (44.9)	<.001
Health care tasks^d^	562 (67.1)	602 (48.2)	<.001
Assists with health care system navigation^e^	539 (63.3)	519 (37.5)	<.001
Frequency of speaking to older adult’s clinician in past year			
Never	321 (43.2)	673 (65.8)	<.001
Rarely	176 (17.5)	91 (5.8)	
Sometimes or often	298 (39.3)	343 (28.4)	
**Older adult characteristics**			
Age, mean (SD), y	78.8 (0.50)	80.4 (0.42)	.009
Female	555 (64.1)	777 (68.1)	.70
Race/ethnicity			
White	429 (68.1)	675 (74.5)	.09
Black	284 (16.3)	339 (13.2)	
Other	83 (15.6)	95 (12.3)	
Enrolled in Medicaid	217 (25.6)	257 (20.2)	.09
Probable dementia	259 (32.5)	354 (24.4)	.65
Self-reported health status			
Excellent or very good	108 (14.3)	260 (26.1)	<.001
Good	256 (33.7)	416 (37.2)	
Fair or poor	432 (52.0)	431 (36.7)	

^a^ Data are from the 2017 National Health and Aging Trends Study and the National Study of Caregiving (unweighted, n = 1905; weighted, n = 17 268 357).

^b^ Percentages are based on data in each column.

^c^ Weighted tests of difference between groups.

^d^ Helped with managing medications, performing medical tasks, and administering shots and/or wound care.

^e^ Helped with making health care appointments and/or coordinating care for older adult.

Among caregivers who assisted during a posthospitalization care transition, 490 (59.1%) reported receiving adequate transitional care training (Table 2). Caregivers who did not report receiving adequate training were more likely to be Black (117 [22.6%] vs 163 [13.9%]; *P* = .03); to report financial difficulty (67 [24.1%] vs 87 [14.4%]; *P* = .008); and to provide care for an older adult who was female (227 [73.2%] vs 316 [62.3%]; *P* = .02), Black (121 [19.8%] vs 163 [14.0%]; *P* = .02) or other non-White race (35 [18.5%] vs 48 [13.5%]; *P* = .02), enrolled in Medicaid (90 [31.9%] vs 127 [21.2%]; *P* = .01), and transitioning to institutional care (75 [30.9%] vs 92 [18.2%]; *P* = .02). Caregivers who did not report receiving adequate training were less likely to be female (180 [50.4%] vs 375 [73.5%]; *P* < .001), to assist with health care system navigation (167 [51.7%] vs 372 [71.3%]; *P* < .001), and to have interacted with the older adult’s clinician sometimes or often (101 [33.9%] vs 197 [43.1%]; *P* < .001) in the past year.

**Table 2.** Characteristics of Family Caregivers Assisting During a Posthospitalization Care Transition by Receipt of Adequate Transitional Care Training^a^

Characteristic	Individuals, No. (%)^b^		*P* value^c^
	Received adequate training (n = 490 [59.1%])	Did not receive adequate training (n = 306 [41.0%])	
**Caregiver characteristics**			
Female	375 (73.5)	180 (50.4)	<.001
Race/ethnicity			
White	269 (69.9)	148 (59.0)	.03
Black	163 (13.9)	117 (22.6)	
Other	58 (16.2)	41 (18.4)	
Educational attainment			
High school or less	187 (38.4)	120 (44.1)	.24
Some college	289 (61.6)	170 (55.9)	
Experiences of burden			
Financial difficulty	87 (14.4)	67 (24.1)	.008
Emotional difficulty	205 (42.6)	139 (39.5)	.56
Physical difficulty	125 (24.3)	75 (19.4)	.21
**Caregiving circumstances**			
Sole caregiver	118 (24.9)	78 (30.6)	.23
Relationship to older adult			
Spouse	113 (26.4)	72 (24.8)	.09
Child	287 (54.4)	153 (45.6)	
Other	90 (19.2)	81 (29.7)	
Paid for caregiving	103 (21.0)	69 (17.2)	.38
Caregiver for ≥5 y	156 (50.0)	85 (57.5)	.21
Caregiving h/mo, mean (SE)	90.8 (9.3)	71.3 (6.7)	.09
Type of assistance			
Mobility	409 (82.8)	267 (90.2)	.02
Personal care	358 (73.1)	212 (71.1)	.53
Health care tasks^d^	360 (69.2)	202 (64.1)	.37
Assists with health system navigation^e^	372 (71.3)	167 (51.7)	<.001
Frequency of speaking to older adult’s clinician in past year			
Never	165 (35.1)	156 (54.8)	<.001
Rarely	127 (21.8)	49 (11.3)	
Sometimes or often	197 (43.1)	101 (33.9)	
**Older adult characteristics**			
Age, mean (SE), y	79.4 (0.54)	78.0 (0.56)	.08
Female	316 (62.3)	227 (73.2)	.02
Race/ethnicity			
White	279 (72.5)	150 (61.7)	.02
Black	163 (14.0)	121 (19.8)	
Other	48 (13.5)	35 (18.5)	
Enrolled in Medicaid	127 (21.2)	90 (31.9)	.01
Probable dementia	167 (26.4)	92 (25.0)	.73
Self-reported health status			
Excellent or very good	71 (15.9)	37 (11.9)	.40
Good	161 (34.6)	95 (32.4)	
Fair or poor	258 (49.5)	174 (55.7)	
Postdischarge destination			
Institution	92 (18.2)	75 (30.9)	.02
Home	395 (81.8)	227 (69.1)	

^a^ Data are from 2017 National Health and Aging Trends Study and the National Study of Caregiving (unweighted, n = 796; weighted, n = 7 083 222).

^b^ Percentages are based on data in each column.

^c^ Weighted tests of difference between groups.

^d^ Helped with managing medications, performing medical tasks, and administering shots and/or wound care.

^e^ Helped with making health care appointments and/or coordinating care for older adult.

Caregivers were half as likely to report receiving adequate transitional care training if they were Black than if they were White (adjusted odds ratio [aOR], 0.52; 95% CI, 0.31-0.89) and if they experienced financial difficulty (aOR, 0.50; 95% CI, 0.31-0.81) (Table 3). Female caregivers were more than twice as likely as male caregivers to report receiving adequate training (aOR, 2.44; 95% CI, 1.65-3.61). Caregivers were significantly more likely to report receiving adequate training if they assisted with health care system navigation (aOR, 2.42; 95% CI, 1.63-3.60) or spoke with the older adult’s clinician about his or her care in the past year sometimes or often (aOR, 1.93; 95% CI, 1.19-3.12) or rarely (aOR, 3.59; 95% CI, 2.03-6.32) vs never.

**Table 3.** Adjusted Odds of Receiving Adequate Transitional Care Training by Characteristics of Family Caregivers Assisting During a Posthospitalization Care Transition^a^

Characteristic	Adjusted odds ratio (95% CI)^b^
Sex	
Male	1 [Reference]
Female	2.44 (1.65-3.61)
Race/ethnicity	
White	1 [Reference]
Black	0.52 (0.31-0.89)
Other	0.78 (0.44-1.37)
Educational attainment	
High school or less	1 [Reference]
Some college	1.20 (0.80-1.79)
Experiences of burden	
Financial difficulty	0.50 (0.31-0.81)
Emotional difficulty	1.13 (0.72-1.80)
Physical difficulty	1.48 (0.91-2.43)
Sole caregiver^c^	0.68 (0.42-1.12)
Relationship to older adult	
Spouse	1 [Reference]
Child	1.29 (0.74-2.24)
Other	0.66 (0.32-1.39)
Paid for caregiving^c^	1.34 (0.75-2.39)
Time spent caregiving, y	
<5	1 [Reference]
≥5	0.65 (0.39-1.08)
Caregiving h/mo	1.00 (0.99-1.00)
Type of assistance^c^	
Mobility	0.58 (0.31-1.09)
Personal care	0.86 (0.57-1.30)
Health care tasks^d^	1.33 (0.82-2.17)
Assists with health care system navigation^e^	2.42 (1.63-3.60)
Frequency of speaking to older adult’s clinician in past year	
Never	1 [Reference]
Rarely	3.59 (2.03-6.32)
Sometimes or often	1.93 (1.19-3.12)

^a^ Data are from the 2017 National Health and Aging Trends Study and the National Study of Caregiving (unweighted, n = 796; weighted, n = 7 083 222).

^b^ Adjusted for older adult age, sex, probable dementia, self-reported overall health status, and postdischarge destination (institution vs home).

^c^ Binary categories for which the reference was not having the condition.

^d^ Helped with managing medications, performing medical tasks, and administering shots and/or wound care.

^e^ Helped with making health care appointments and/or coordinating care for older adult.

## Discussion

To our knowledge, this study provides the first national profile of family and unpaid caregivers who assisted with posthospitalization care transitions, including whether they reported receiving adequate transitional care training. We found that caregivers who assisted with posthospitalization care transitions provided higher-intensity care and were more likely to experience caregiving-related difficulty. Of caregivers who assisted with a posthospitalization care transition, more than half (59.1%) reported receiving adequate transitional care training. However, caregivers were half as likely to report receiving adequate training if they were Black; experienced financial difficulty; interacted infrequently with the health care system; or assisted older adults who were Black, female, or enrolled in Medicaid. Our findings raise concerns regarding potential disparities between groups of individuals who receive adequate transitional care training and highlight the importance of health care system interactions in facilitating training. Our study findings contribute to a larger body of research that has demonstrated greater rates of hospital readmissions among older adults who are non-White or have lower socioeconomic status^37,38^ and suggest an opportunity to reduce adverse events after hospital discharge through systematic assessment and targeted education of family and unpaid caregivers who assist older adults during posthospitalization care transitions.

Caregivers who reported not receiving adequate transitional care training were more likely to be Black; experience financial difficulty; or care for a Black, female, or Medicaid-enrolled older adult. These caregivers may have had lower health literacy levels,^39^ encountered language barriers,^40^ and been less comfortable voicing questions in the hospital, all of which may impede communication with clinicians. In a nationally representative survey, less than half of family caregivers reported being asked whether they needed help managing the older adult’s care by a clinician.^41^ This failure to systematically assess caregivers’ needs as part of discharge planning places the responsibility on caregivers to advocate for themselves to address information gaps related to older adults’ posthospitalization care needs. This may be especially difficult for caregivers who are not White and have lower socioeconomic status and/or health literacy. These caregivers report interacting less frequently with clinicians,^41^ are most at risk for imbalanced interactions with clinicians,^42,43^ and often report feeling invisible in the hospital environment.^40^

Disparities in receipt of adequate training may also be associated with structural racism and implicit biases^44,45^ affecting health care professionals’ evaluations of which caregivers need training and/or are likely to comply with training instructions. Standardized caregiver assessment instruments are rarely used in acute care settings,^46,47^ and little is known regarding the cultural relevance and appropriateness of available assessments.^48^ Instead, clinicians’ individual judgments, based on limited interactions under substantial time pressure, determine whether caregivers receive training.^46^ The use of a standardized, culturally relevant instrument to assess caregivers’ need for training as part of the discharge process may be associated with reduced possibility of bias and the expectation that caregivers advocate for their own training while helping clinicians to quickly and effectively determine caregivers’ education needs related to postdischarge care.^46,49^ To inform development of such an instrument and appropriate training resources, qualitative research is needed to elucidate caregiver perspectives regarding inadequate aspects of training and the most concerning knowledge gaps that they face in providing posthospitalization care to older adults.

Our finding that caregivers’ interactions with the health care system were associated with receiving adequate transitional care training underscores the critical role of clinicians in educating caregivers and supporting high-quality care delivery. Fostering clinician-caregiver partnerships is challenging given the absence of supportive reimbursement opportunities and limited time available for clinician-caregiver interactions,^46,50^ especially during the discharge process.^51,52,53^ Widespread limitations on hospital visitation in the wake of the coronavirus disease 2019 (COVID-19) pandemic have likely further inhibited caregivers’ access to adequate transitional care training because caregivers have fewer opportunities to directly interact with clinicians.^54^

We observed that caregivers who assisted older adults during posthospitalization care transitions were more likely to report physical, emotional, and financial difficulty associated with caregiving, echoing previous findings that assisting during a care transition is especially burdensome.^31,55,56^ Therefore, it is promising that recent policies have been designed to encourage health care professional–led support of caregivers during the discharge process. A 2017 revision to the Medicare Hospital Conditions of Participation requires counseling of patients and family members to prepare them for posthospitalization care.^57^ The CARE Act requires hospitals to provide education related to postdischarge medical tasks,^34,58^ although implementation has varied widely by state and includes few enforcement mechanisms.^59,60^ Although engaging family caregivers during discharge may be associated with improved postdischarge outcomes,^21^ the effects of these policies on caregivers are not well understood. Future evaluation of these policies and programs should examine whether they impact caregiver capacity and burden.

### Limitations

This study has limitations. Information regarding the mode of transitional care training or its effects on caregiver burden or capacity was not available. Similarly, we did not have access to granular information regarding older adults’ specific care settings after hospital discharge (eg, skilled nursing, inpatient rehabilitation, or home health care). Given the use of cross-sectional, observational data, causal inferences could not be made. A small number of study participants reported primary race/ethnicities other than White or Black. As a result, we could not evaluate the unique experiences of these caregivers and how they varied; this is a topic that deserves further attention. We did not have access to information regarding the older adults’ hospital stays, including diagnoses, duration of the hospital stays, or characteristics of hospitals where care was received. Our estimate of caregiver access to transitional care training is based on data gathered before the COVID-19 pandemic; the current rate of training is likely lower.

## Conclusions

In this cross-sectional and nationally representative study of older adults’ family caregivers, assisting during a posthospitalization care transition was associated with providing more intensive care and a greater likelihood of caregivers reporting difficulty related to the role. More than half (59.1%) of caregivers who helped an older adult during a posthospitalization care transition in the previous year reported receiving the training that they needed from hospital staff. Caregivers were more likely to report receiving adequate transitional care training if they interacted frequently with the health care system, whereas such training was less likely if caregivers were Black; experienced financial difficulty; or cared for a Black, female, or Medicaid-enrolled older adult. These findings suggest the need for greater support of family caregivers who assist older adults during posthospitalization care transitions and the need for formal, standardized assessment tools to help clinicians identify which caregivers may benefit from transitional care training.
